# Editorial: Interoception and the autonomic nervous system: Investigating affect, decision-making, and mental health

**DOI:** 10.3389/fnins.2022.1130324

**Published:** 2023-01-13

**Authors:** Daisuke Ueno, Hideki Ohira, Jin Narumoto

**Affiliations:** ^1^Department of Psychiatry, Graduate School of Medical Science, Kyoto Prefectural University of Medicine, Kyoto, Japan; ^2^Department of Psychology, Graduate School of Informatics, Nagoya University, Nagoya, Japan

**Keywords:** interoception, vagus nerve, time perception, value updating, Alzheimer's disease (AD), heartbeat perception training, anxiety, depression

The concept of interoception has been expanded to include not only the process of control in which the bodily state is altered in response to environmental demands, but also the perception of bodily states by afferent processing while maintaining homeostasis or allostasis, the activity of the vagus nerve, neuroendocrine, and immune system (Chen et al., 2021). Interoception was first described by Sir Charles Scott Sherrington, who was a British Physiologist and Nobel Prize winner for discoveries about the function of neurons in 1932 with 1st Baron Edgar Douglas Adrian (Sherrington, 1906). Although the autonomic nervous system has been defined as efferent fibers (Langley, 1898), Prechtl and Powley (1990) discovered lamina-I neurons as the afferent pathway of interoception, including temperature, pain, and visceral sensations. Craig (2002) stated that the interoception transmitted by lamina-I neurons elicits autonomic activity; it has been shown that interoception constructs emotions.

In this Research Topic, each paper contributes to how interoception and the autonomic nervous system (ANS), especially the vagus nerve, correlate with each other and how understanding the relationship between interoception and the autonomic nervous system helps us understand how the body adapts to a complex environment. Interoception plays a role in energy regulation, feeling, emotional experience, and the sense of self by monitoring and regulating one's bodily state (Quigley et al., 2021). In other words, interoception maintains homeostasis or allostasis to adapt the body to a complex environment.

Uraguchi et al. demonstrated that higher interoceptive accuracy (IAc) is correlated with less dispersion of the perceived duration and higher temporal sensitivity, but with less deviation of the perceived duration from the real duration in an easy temporal precision task. In difficult temporal tasks in complex environments, interoceptive accuracy is not correlated with temporal perception. Tomyta et al. demonstrated that higher IAc is correlated with less timing control in difficult conditions using rhythmic synchronization tapping with metronome sounds. Thus, in complex external conditions, interoception decreases confidence in time prediction but not in less complex external conditions. Moreover, in complex external conditions, it is difficult to predict the external environment from interoception alone, a higher IAc becomes a distractor, and interoceptive signals must be suppressed to control exteroceptive signals.

Kimura et al. demonstrated that the learning rate for positive prediction errors was higher than that for negative prediction errors in systole trials; however, the learning rates did not differ between positive and negative prediction errors in diastole trials. In other words, the natural fluctuation of cardiac afferent signals can affect asymmetric value updating in reward learning, and cardiac interoceptive signals can modulate decision making.

The clarification of interoception and the autonomic nervous system will not only deepen our understanding of human perception and cognition, but will also suggest new clinical pictures and treatments for degenerative diseases such as Alzheimer's disease (AD) and psychiatric symptoms such as anxiety and depression.

Sun et al. highlighted that the decrease in decision-making in patients with Alzheimer's disease (AD) is possibly caused by disconnecting the IAc and interoceptive awareness, and this disconnection causes anosognosia of amnesia or disorientation. Sun et al. hypothesized that the discrepancy between objectively and subjectively perceived physical states causes homeostasis to break down, resulting in decreased decision-making capacity.

Flux et al. demonstrated that floatation-REST (Reduced Environmental Stimulation Therapy) relative to the film-watching condition elicited decreases in blood pressure, breathing rate, and the sympathetic nervous system by HRV parameters, but elicited increases in the parasympathetic nervous system by HRV parameters across both anxious and non-anxious participants. In other words, reduced environmental stimulation (exteroception), enhances interoception, lowers sympathetic arousal, and alters the balance of the autonomic nervous system toward a more parasympathetic state.

Schillings et al. demonstrated that heartbeat perception training once a week enhances IAc in the short-term but not interoceptive confidence (IS) in the short term and IAc and IS in 3 weeks. Although heartbeat perception training is one of the means to improve IAc, it is significant to note from Schillings et al. that training once a week for 20 min had an initial short-term effect but no longitudinal effect over a 3-week period.

Tan et al. highlighted that the vagus nerve serves as a bidirectional communication pathway between the gut and the brain through the vagus afferent and efferent fibers and plays an important role in the onset and deterioration of depression. Moreover, Tan et al. hypothesized that restoration of the disturbance of the microbiota-gut-brain axis by the administration of probiotics or stimulation of the vagus nerve, electrically or chemically, may alleviate depressive symptoms by improving gut inflammation and permeability.

In summarizing these articles, it is evident that interoception contributes to the maintenance and regulation of environmental demands in the individual phenomena of time perception, decision making, as well as emotional and psychiatric symptoms through the vagus nerve. In closing, we would like to convey our gratitude to all those who have undertaken the writing of the papers, served as editors, reviewers, and the editorial office under these circumstances.

## Author contributions

DU, HO, and JN have written this editorial for the Research Topic they have edited. All authors contributed to the article and approved the submitted version.
